# Heterogeneity in outcome selection, definition and measurement in studies assessing the treatment of cryptoglandular anal fistula: findings from a systematic review

**DOI:** 10.1007/s10151-021-02452-5

**Published:** 2021-05-08

**Authors:** A. J. H. M. Machielsen, N. Iqbal, M. L. Kimman, K. Sahnan, S. O. Adegbola, G. Kane, R. Woodcock, J. Kleijnen, U. Grossi, S. O. Breukink, P. J. Tozer

**Affiliations:** 1 Department of Surgery and Colorectal Surgery, Maastricht University Medical Centre+, Maastricht, The Netherlands; 2 Faculty of Health, Medicine and Life Sciences, Maastricht University, Maastricht, The Netherlands; 3 Robin Phillips’ Fistula Research Unit, St Mark’s Hospital, London, UK; 4 Department of Surgery and Cancer, Imperial College London, London, UK; 5 Department of Clinical Epidemiology and Medical Technology Assessment, Care and Public Health Research Institute (CAPHRI), Maastricht University Medical Centre+, Maastricht, The Netherlands; 6 Belfast, UK; 7 London, UK; 8 Department of Family Medicine, Care and Public Health Research Institute (CAPHRI), Maastricht University, Maastricht, The Netherlands; 9 Tertiary Referral Pelvic Floor and Incontinence Centre, Regional Hospital Treviso, Treviso, Italy; 10 Department of Surgery and Colorectal Surgery, Nutrim School of Nutrition and Translational Research in Metabolism, Maastricht University Medical Centre+, Grow School for Oncology and Developmental Biology, Maastricht, The Netherlands

**Keywords:** Cryptoglandular anal fistula, Systematic review, Outcome assessment, Core outcome set

## Abstract

**Background:**

Treatment for cryptoglandular anal fistula (AF) is challenging and a lack of uniform outcomes in the literature prevents direct comparison of treatments. This can be addressed by developing a core outcome set, a standardised set of outcomes reported in all interventional studies for a specific condition. The aim of this systematic review is to assess the range of outcomes, their definitions, and the measurement instruments currently utilised in interventional studies for adult patients with AF. This will inform the development of an AF core outcome set.

**Methods:**

Medline, Embase and The Cochrane Library were searched to identify all patient- and clinician-reported outcomes in studies assessing medical, surgical or combination treatment of adult patients with AF published from January 2008 to May 2020. The resulting outcomes were categorized according to the Core Outcome Measurement in Effectiveness Trials (COMET) taxonomy to better understand their distribution.

**Results:**

In total, 155 studies were included, 552 outcomes were extracted, with a median of three outcomes (interquartile range 2–5) per study. Only 25% of studies demonstrated high-quality outcome reporting. The outcomes were merged into 52 unique outcomes and structured into four core areas and 14 domains, with the majority in the domain of physiological or clinical (gastrointestinal) outcomes. The most commonly reported outcomes were healing (77%), incontinence (63%), and recurrence (40%), with no single outcome assessed across all studies. There was a wide variation in outcome definitions and measurement instruments used.

**Conclusions:**

There is substantial heterogeneity in outcomes, definitions, and measurement instruments reported in interventional studies for cryptoglandular anal fistula. This emphasises the need for standardised outcome reporting and measurement.

## Introduction

Cryptoglandular anal fistula (AF) is a challenging condition to manage. The symptom burden can be severe and can have wide-ranging impact on physical functioning and quality of life [1]. For clinicians, the difficulties of balancing treatment efficacy with minimal impairment of continence have been well documented [2, 3], particularly for complex and recurrent cases. In an attempt to address the dichotomy in achieving these key treatment aims, numerous sphincter-preserving procedures have been developed in recent decades. These procedures have now made their way into common clinical practice, leading to wide variation in the techniques used according to surgical expertise, preference, and geographical area [4]. Along with the expansion of procedures, numerous interventional studies have been conducted to assess success rates and determine treatment superiority. Attempts have been made to meta-analyse data from multiple studies, however, difficulties in doing so reliably are frequently reported, due to inadequate follow-up, lack of randomized controlled trials, and non-uniform reporting of outcomes [4–6]. This limits the development of treatment guidelines for AF.

The selection of relevant and appropriate outcomes is crucial to any study on treatment effectiveness [7], however, the lack of a systematic approach results in the reporting of numerous outcomes with varied definitions, multiple measurement instruments, and inconsistencies in the timing of assessment. Furthermore, selective reporting of outcomes based on significant results is a recognised problem and can overestimate the size of the treatment effect [8, 9]. Such outcome reporting bias can lead to ill-informed decisions with the potential to cause patient harm [10].

One way of addressing such issues is to develop a core outcome set (COS); an agreed, standardised set of outcomes to be measured in all interventional studies for a specific health condition [9]. The importance and value of a COS in disease areas with heterogeneity in outcome reporting is being increasingly recognised. However, a COS has not yet been developed for cryptoglandular AF. We believe that this is an important step in addressing the challenges in developing evidence-based management strategies.

According to the Core Outcome Measurement in Effectiveness Trials (COMET) initiative, the first stage in the development of a COS is to determine what to measure, which can be partially achieved by identifying potential outcomes from the existing literature [7]. The primary aim of this systematic review was to identify all patient- and clinician-reported outcomes in studies assessing medical, surgical or combination treatment of adult patients with cryptoglandular AF, to inform the development of a cryptoglandular Anal Fistula Core Outcome Set (AFCOS) [11]. The secondary aim is to assess outcome definitions and identify the measurement instruments used.

## Materials and methods

A systematic review of studies assessing medical, surgical, and combined interventions for cryptoglandular AF was performed in accordance with a registered protocol (PROSPERO-ID CRD42018102778).

### Search strategy

An electronic search strategy was developed by an information specialist prior to execution. The following electronic databases were searched, adjusting vocabulary and syntax for each: Medline (Ovid), Embase (Ovid), and The Cochrane Library. Validated terms for ‘Perianal Fistula’ were used, ensuring that all interventional studies for AF could be captured. If MeSH terms or subject headings existed, these were included in the search strategy and supplemented with free-text searches of the same databases. To avoid limiting the scope of outcomes identified, no study design filter was applied. The search was restricted to full-text articles in English published from January 2008 to May 2020 and to studies conducted in human subjects aged ≥ 18 years. The full search strategy can be found in Table 1.

**Table 1:** Search strategy

Embase (Ovid)	
1	Anus fistula/
2	Rectum fistula/
3	((Anus or anal or anorectal or rectal or rectum or perianal) adj4 fistul*).m_titl
4	Fistula ani.m_titl
5	Fistula-in-ano.m_titl
6	1 or 2 or 3 or 4 or 5
7	Limit 6 to (full text and human and year = “2008–current”)
The Cochrane library	
1	MeSH descriptor: (rectal fistula) this term only
2	(Anus or anal or anorectal or rectal or rectum or perianal) near/4 (fistul*):ti
3	Fistula ani:ti
4	Fistula-in-ano:ti
5	#1 or #2 or #3 or #4
	Publication date from January 2008 to May 2020
Medline (Ovid)	
1	Rectal fistula/
2	((Anus or anal or anorectal or rectal or rectum or perianal) adj4 fistul*).m_titl
3	Fistula ani.m_titl
4	Fistula-in-ano.m_titl
5	1 or 2 or 3 or 4
6	Limit 5 to (full text and humans and year = “2008–current”)

### Study selection

Four members of the study management group (AM, NI, KS, SA) identified and screened titles and abstracts using Covidence Systematic Review Software (Veritas Health Innovation, Melbourne, Australia, available at https://www.covidence.org/home), with each abstract and full-text publication screened by two independent group members. The following predefined selection criteria were used: (1) Prospective [including randomised controlled trials (RCTs), cohort comparisons, case controls and case series], retrospective, and observational studies including ≥ 10 patients and systematic reviews published between January 2008 and May 2020; (2) including ≥ 10 adult patients (aged ≥ 18 years) with cryptoglandular AF; (3) assessing medical, surgical, or combined interventions for cryptoglandular AF; (4) and reporting ≥ one outcome. Studies were excluded if they were abstract only or if they reported on interventions that were only assessed on fistulas that were not perianal or not of cryptoglandular origin. Systematic reviews were included and individual studies were checked for eligibility. Disagreements were resolved through discussion with recourse to the senior authors (PT, SB) if necessary.

### Data extraction

Two members of the study management group (AM, NI) extracted data from eligible studies using a predefined data extraction sheet created in Microsoft Excel. Extracted data included study publication year, design, interventions, patients, outcomes (primary and secondary), outcome definitions and measurement instruments used. In keeping with COMET recommendations, all data were extracted verbatim [7]. The quality of describing and reporting outcomes was assessed using Harman’s criteria [12], which are presented in (Table 2). Disagreements were resolved through discussion with recourse to the senior authors (PT, SB) if necessary.

**Table 2:** Overview of the included studies

First author	Title	Year of publication	Study design	Intervention(s)	Number of participants	Number of outcome(s)	Primary outcome clearly stated?	Primary outcome clearly defined?	Secondary outcomes clearly stated?	Secondary outcomes clearly defined?	The use of the selected outcomes explained?	Methods used to enhance the quality of outcome measurement?
A ba-bai-ke-re	Randomized controlled trial of minimally invasive surgery using acellular dermal matrix for complex anorectal fistula	2010	RCT	ADM ERAF	90	6	Yes	Yes	Yes	Yes	No	Yes
Abcarian	Ligation of intersphincteric fistula tract: early results of a pilot study	2012	Prospective study	LIFT procedure	40	3	Yes	No	Yes	No	No	No
Abdelnaby	Drained mucosal advancement flap versus rerouting seton around the internal anal sphincter in treatment of high trans-sphincteric anal fistula: a randomized trial	2019	RCT	Advancement flap and drainage seton around EAS Seton around IAS	97	6	Yes	Yes	Yes	Yes	No	No
Aboulian	Early result of ligation of the intersphincteric fistula tract for fistula-in-ano	2011	Retrospective review	LIFT procedure	25	5	Yes	No	Yes	No	No	No
Adams	Long-term outlook after successful fibrin glue ablation of cryptoglandular transsphincteric fistula-in-ano	2008	Retrospective review	Fibrin glue	36	2	Yes	No	Yes	No	No	No
Adamina	To plug or not to plug: a cost-effectiveness analysis for complex anal fistula	2010	Prospective study	AFP ERAF	24	2	Yes	Yes	N/A	N/A	Yes	No
Altomare	Seton or glue for trans-sphincteric anal fistulae: a prospective randomized crossover clinical trial	2011	RCT	Fibrin glue Seton	64	5	Yes	No	Yes	Yes	No	No
Alvandipour	Efficacy of 10% sucralfate ointment after anal fistulotomy: a prospective, double-blind, randomized, placebo-controlled trial	2016	RCT	Fistulotomy and 10% sucralfate Fistulotomy and placebo	41	2	Yes	Yes	Yes	Yes	No	No
Anan	Fistulotomy with or without marsupialisation of wound edges in treatment of simple anal fistula: a randomised controlled trial	2019	RCT	Fistulotomy Fistulotomy and marsupialization	60	5	Yes	Yes	Yes	Yes	No	No
Arawatti	Standardization and clinical evaluation of nimba ksharsutra in the management of bhagandar (fistula in ano)	2012	RCT	Seton Seton	40	5	Yes	Yes	No	N/A	No	No
Arroyo	Photodynamic therapy for the treatment of complex anal fistula	2017	Prospective study	Photodynamic therapy	10	4	Yes	Yes	Yes	Yes	No	No
Arroyo	Fistulotomy and sphincter reconstruction in the treatment of complex fistula-in-ano: long-term clinical and manometric results	2012	Prospective study	FISR	70	2	Yes	No	Yes	No	No	Yes
Atkin	For many high anal fistulas, lay open is still a good option	2011	Retrospective review	EUA Drainage of abscess Fistulotomy (and marsupialization) Seton Fibrin glue Advancement flap Transperineal core-out and repair Martius flap Sphincter repair Defunctioning stoma Proctectomy and permanent colostomy	180	3	Yes	No	Yes	No	No	No
Attaallah	Should we consider topical silver nitrate irrigation as a definitive nonsurgical treatment for perianal fistula	2014	Prospective study	Irrigation and 1% silver nitrate	56	3	Yes	Yes	Yes	Yes	No	Yes
Bleier	Ligation of the intersphincteric fistula tract: an effective new technique for complex fistulas	2010	Retrospective review	LIFT procedure	39	3	Yes	No	Yes	No	No	No
Boenicke	Advancement flap for treatment of complex cryptoglandular anal fistula: prediction of therapy success or failure using anamnestic and clinical parameters	2017	Prospective study	Advancement flap	61	2	Yes	Yes	Yes	Yes	No	No
Bondi	Randomized clinical trial comparing collagen plug and advancement flap for transsphincteric anal fistula	2017	RCT	AFP Advancement flap	94	4	Yes	Yes	Yes	Yes	No	Yes
Browder	Modified Hanley procedure for management of complex horseshoe fistulae	2009	Retrospective review	Modified Hanley procedure, drainage and setons	23	4	Yes	No	Yes	No	No	No
Chalya	Fistulectomy versus fistulotomy with marsupialisation in the treatment of low fistula-in-ano: a prospective randomized controlled trial	2013	RCT	Fistulectomy Fistulotomy and marsupialization	162	9	Yes	Yes	Yes	Yes	No	No
Chan	Initial experience of treating anal fistula with the Surgisis anal fistula plug	2012	Prospective study	AFP	44	2	Yes	Yes	Yes	No	No	No
Chen	High ligation of the fistula track by lateral approach: a modified sphincter-saving technique for advanced anal fistulas	2012	Prospective study	Modified LIFT procedure	10	4	No	N/A	No	N/A	Yes	No
Chowbey	Minimally invasive anal fistula treatment (MAFT)—an appraisal of early results in 416 patients	2015	Prospective study	MAFT	416	3	Yes	No	No	N/A	No	No
Chung	Anal fistula plug and fibrin glue versus conventional treatment in repair of complex anal fistulas	2009	Retrospective review	AFP Fibrin glue Advancement flap Seton	232	1	Yes	Yes	N/A	N/A	No	No
Choi	Patient-performed seton irrigation for the treatment of deep horseshoe fistula	2010	Retrospective review	Seton Seton	24	4	Yes	Yes	No	N/A	No	No
Choi	Autologous adipose tissue-derived stem cells for the treatment of complex perianal fistulas not associated with Crohn’s disease: a phase II clinical trial for safety and efficacy	2017	Prospective study	ASC	15	5	Yes	Yes	Yes	Yes	No	Yes
Christoforidis	Treatment of complex anal fistulas with the collagen fistula plug	2008	Retrospective review	AFP	47	1	Yes	No	N/A	N/A	No	No
Christoforidis	Treatment of transsphincteric anal fistulas by endorectal advancement flap or collagen fistula plug: a comparative study	2009	Retrospective review	ERAFAFP	80	3	Yes	Yes	Yes	Yes	No	No
Cintron	Treatment of fistula-in-ano using a porcine small intestinal submucosa anal fistula plug	2013	Prospective study	AFP	73	2	Yes	Yes	Yes	Yes	No	No
Daodu	Draining setons as definitive management of fistula-in-ano	2018	Retrospective study	Seton	76	2	Yes	Yes	No	N/A	No	No
De La Portilla	Platelet-rich plasma (PRP) versus fibrin glue in cryptogenic fistula-in-ano: a phase III single-center, randomized, double-blind trial	2019	RCT	PRP Fibrin glue	56	5	Yes	Yes	Yes	No	No	Yes
De La Portilla	Treatment of transsphincteric fistula-in-ano with growth factors from autologous platelets: results of a phase II clinical trial	2017	Prospective study	PRGF	36	5	Yes	Yes	No	N/A	No	No
De La Portilla	Evaluation of a new synthetic plug in the treatment of anal fistulas: results of a pilot study	2011	Prospective study	AFP	19	4	Yes	No	No	N/A	No	No
Dubsky	Endorectal advancement flaps in the treatment of high anal fistula of cryptoglandular origin: full thickness vs mucosal rectum flaps	2008	Retrospective review	Advancement flap Advancement flap	54	2	No	N/A	No	N/A	No	No
Dozois	Early results of a phase I trial using an adipose-derived mesenchymal stem cell-coated fistula plug for the treatment of transsphincteric cryptoglandular fistulas	2019	Prospective study	AFP	15	3	Yes	No	Yes	Yes	No	Yes
Ege	Hybrid seton for the treatment of high anal fistulas: results of 128 consecutive patients	2014	Retrospective review	Seton	128	5	Yes	No	No	N/A	No	No
Eitan	The use of the loose seton technique as a definitive treatment for recurrent and persistent high trans-sphincteric anal fistulas: a long-term outcome	2009	Retrospective review	Seton	41	3	Yes	Yes	No	N/A	No	No
Ellis	Outcomes with the use of bioprosthetic grafts to reinforce the ligation of the intersphincteric fistula tract (BioLIFT procedure) for the management of complex anal fistulas	2010	Retrospective review	BioLIFT procedure	31	2	Yes	Yes	N/A	N/A	No	No
Ellis	Long-term outcomes with the use of bioprosthetic plugs for the management of complex anal fistulas	2010	Retrospective review	AFP	63	1	Yes	No	No	N/A	No	No
Fabiani	Permacol collagen paste injection for the treatment of complex anal fistula: 1-year follow-up	2017	Prospective study	Collagen paste injection	21	3	Yes	No	Yes	No	No	No
Fung	Operative strategy for fistula-in-ano without diversion of the anal sphincter	2013	Retrospective review	Partial fistulotomy and seton	46	3	Yes	Yes	Yes	Yes	No	Yes
Garcia-Arranz	Autologous adipose-derived stem cells for the treatment of complex cryptoglandular perianal fistula: a randomized clinical trial with long-term follow-up	2020	RCT	ASC and fibrin glue Fibrin glue	57	4	Yes	Yes	N/A	N/A	No	Yes
Garcia-Olmo	Expanded adipose-derived stem cells for the treatment of complex perianal fistula: a phase II clinical trial	2009	RCT	Fibrin glueFibrin glue and ASC	49	4	Yes	No	No	N/A	No	No
Garg	PERFACT procedure (Proximal superficial cauterization, emptying regularly of fistula tracts and curettage of tracts): A new concept to treat highly complex anal fistula	2015	Prospective study	PERFACT procedure	51	3	Yes	No	No	N/A	No	No
Garg	To determine the efficacy of anal fistula plug in the treatment of high fistula-in-ano: an initial experience	2009	Prospective study	AFP	21	7	No	N/A	No	N/A	No	No
Gautier	Easy clip to treat anal fistula tracts: a word of caution	2015	Retrospective study	Clip	17	10	Yes	No	No	N/A	No	No
Giamundo	Fistula-tract laser closure (FiLaC): long-term results and new operative strategies	2015	Retrospective study	FiLaC	45	5	Yes	Yes	Yes	Yes	No	No
Gottgens	Ligation of the intersphincteric fistula tract for high transsphincteric fistula yields moderate results at best: is the tide turning?	2019	Retrospective study	LIFT BioLIFT	46	5	Yes	Yes	Yes	No	No	No
Gottgens	Long-term results of mucosal advancement flap combined with platelet-rich plasma for high cryptoglandular perianal fistulas	2014	Retrospective study	Advancement flap and platelet-rich plasma	25	2	Yes	Yes	Yes	Yes	No	No
Grolich	Role of video-assisted anal fistula treatment in our management of fistula-in-ano	2014	Retrospective review	VAAFT	30	2	Yes	No	No	N/A	No	No
Gupta	Topical sucralfate treatment of anal fistulotomy wounds: a randomized placebo-controlled trial	2011	RCT	Fistulotomy and 7% sucralfate Fistulotomy and placebo	80	3	Yes	Yes	Yes	Yes	No	No
Haim	Long-term results of fibrin glue treatment for cryptogenic perianal fistulas: a multicenter study	2011	Retrospective review	Fibrin glue	23	3	Yes	Yes	Yes	Yes	No	Yes
Hall	Outcomes after operations for anal fistula: results of a prospective, multicenter, regional study	2014	Retrospective review	Fistulotomy LIFT procedure Seton Advancement flap AFP	240	2	Yes	No	No	N/A	No	No
Hammond	Management of idiopathic anal fistula using cross-linked collagen: a prospective phase 1 study	2011	Prospective study	Collagen paste injection Fibrin glue	29	6	Yes	Yes	Yes	Yes	No	No
Han	Ligation of intersphincteric fistula tract vs ligation of the intersphincteric fistula tract plus a bioprosthetic anal fistula plug procedure in patients with transsphincteric anal fistula: early results of a multicenter prospective randomized trial	2016	RCT	LIFT procedure LIPT-plug procedure	237	5	Yes	Yes	No	N/A	No	No
Han	Long-term outcomes of human acellular dermal matrix plug in closure of complex anal fistulas with a single tract	2011	Retrospective study	ADM	114	2	Yes	Yes	No	N/A	No	No
Han	Ligation of the intersphincteric fistula tract plus bioprosthetic anal fistula plug (LIFT-Plug): a new technique for fistula-in-ano	2013	Prospective study	LIFT-plug procedure	21	3	Yes	Yes	Yes	Yes	No	No
Herold	Results of the Gore Bio-a fistula plug implantation in the treatment of anal fistula: a multicentre study	2016	Prospective study	AFP	60	6	Yes	Yes	Yes	Yes	No	Yes
Herreros	Autologous expanded adipose-derived stem cells for the treatment of complex cryptoglandular perianal fistulas: a phase III randomized clinical trial (FATT 1: fistula advanced therapy trial 1) and long-term evaluation	2012	RCT	ASC ASC and fibrin glue Fibrin glue	183	4	Yes	Yes	No	N/A	No	No
Hirschburger	Fistulectomy with primary sphincter reconstruction in the treatment of high transsphincteric anal fistulas	2014	Retrospective review	FISR	50	4	No	N/A	No	N/A	No	No
Han	Ligation of the intersphincteric fistula tract plus a bioprosthetic anal fistula plug (LIFT‐plug): a new technique for fistula‐in‐ano	2013	Prospective study	LIFT-plug procedure	46	5	No	N/A	Yes	No	No	No
Heydari	Bioabsorbable synthetic plug in the treatment of anal fistulas	2013	Retrospective review	AFP	48	3	Yes	No	No	N/A	No	No
Hyman	Outcomes after fistulotomy: results of a prospective, multicenter regional study	2009	Prospective study	Fistulotomy Seton Fistulotomy AFP Fibrin glue Advancement flap	245	3	Yes	Yes	Yes	Yes	No	No
Jain	Comparison of a fistulectomy and a fistulotomy with marsupialization in the management of a simple anal fistula: a randomized, controlled pilot trial	2012	RCT	Fistulectomy Fistulotomy and marsupialization	40	9	Yes	No	No	N/A	No	No
Jivapaisarnpong	Core out fistulectomy, anal sphincter reconstruction and primary repair of internal opening in the treatment of complex anal fistula	2009	Prospective study	FISR	33	4	No	N/A	No	N/A	No	No
Jarrar	Advancement flap repair: a good option for complex anorectal fistulas	2011	Retrospective study	Advancement flap	98	3	Yes	No	Yes	No	No	No
Jayne	Anal fistula plug versus surgeon’s preference for surgery for transsphincteric anal fistula: the FIAT RCT	2019	RCT	AFP Surgeon’s preference (fistulotomy, seton, advancement flap or LIFT)	304	6	Yes	Yes	Yes	No	Yes	No
Jiang	Video-assisted anal fistula treatment (VAAFT) for complex anal fistula: a preliminary evaluation in China	2017	Retrospective review	VAAFT	52	4	Yes	Yes	No	N/A	No	No
Kalim	Comparison of mean healing time and mean pain scores between fistulectomy and fistulotomy for the treatment of low fistula in ano	2017	RCT	Fistulectomy Fistulotomy	304	3	Yes	No	Yes	No	No	No
Ky	Collagen fistula plug for the treatment of anal fistulas	2008	Prospective study	AFP	45	3	Yes	Yes	No	N/A	No	No
Kelly	The role of loose seton in the management of anal fistula: a multicenter study of 200 patients	2014	Retrospective review	Seton	200	3	Yes	No	No	N/A	No	No
Khafagy	Treatment of anal fistulas by partial rectal wall advancement flap or mucosal advancement flap: a prospective randomized study	2010	Prospective study	Advancement flap Advancement flap	40	5	Yes	No	No	N/A	No	No
Kochhar	Video-assisted anal fistula treatment	2014	Retrospective review	VAAFT	82	5	Yes	Yes	Yes	Yes	No	No
Lara	Platelet-rich fibrin sealant as a treatment for complex perianal fistulas: a multicentre study	2015	Prospective study	PRF	60	3	Yes	Yes	No	N/A	No	Yes
Lawes	Early experience with the bioabsorbable anal fistula plug	2008	Retrospective review	AFP	20	2	Yes	No	No	N/A	No	No
Leventoglu	Treatment for horseshoe fistula with the modified Hanley procedure using a hybrid seton: results of 21 cases	2013	Prospective study	Modified Hanley procedure	21	7	Yes	No	Yes	No	No	Yes
Liu	Long-term results of ligation of intersphincteric fistula tract (LIFT) for fistula-in-ano	2013	Retrospective review	LIFT procedure	38	5	Yes	Yes	No	N/A	No	No
Lupinacci	Treatment of fistula-in-ano with the Surgisis AFP anal fistula plug	2010	Prospective study	AFP	15	2	Yes	Yes	Yes	Yes	No	No
Lo	Ligation of intersphincteric fistula tract procedure for the management of cryptoglandular anal fistulas	2012	Prospective study	LIFT procedure	25	5	Yes	Yes	No	N/A	No	No
Lehman	Efficacy of LIFT for recurrent anal fistula	2013	Prospective study	LIFT procedure	17	2	No	N/A	No	N/A	Yes	No
Lobo	A comparative clinical study of Snuhi Ksheera Sutra, Tilanala Kshara Sutra and Apamarga Kshara Sutra in Bhagandara (fistula in ano)	2012	Prospective study	Seton Seton Seton	33	3	Yes	Yes	No	N/A	No	No
Madbouly	Ligation of intersphincteric fistula tract versus mucosal advancement flap in patients with high transsphincteric fistula-in-ano: a prospective randomized trial	2014	RCT	LIFT procedure Advancement flap	70	6	Yes	Yes	Yes	No	No	No
Malakorn	Ligation of intersphincteric fistula tract for fistula in ano: lessons learned from a decade of experience	2017	Retrospective review	LIFT procedure	251	1	Yes	Yes	N/A	N/A	No	No
Mansour	Medical interventional treatment of adult fistula-in-ano. A pilot study for curative response of intra-tract injections of Ceftazidine and Metronidazol	2016	RCT	Ceftazidime and Metronidazole injection	25	2	No	N/A	No	N/A	No	No
Mascagni	OTSC proctology vs. fistulectomy and primary sphincter reconstruction as a treatment for low trans-sphincteric anal fistula in a randomized controlled pilot trial	2019	Retrospective study	Clip Fistulectomy	30	3	No	N/A	No	N/A	No	No
Mascagni	Total fistulectomy, sphincteroplasty and closure of the residual cavity for transsphincteric perianal fistula in the elderly patient	2017	Retrospective review	FISR	86	6	No	N/A	No	N/A	No	No
McGee	Tract length predicts successful closure with anal fistula plug in cryptoglandular fistulas	2010	Prospective study	AFP	41	2	Yes	No	No	N/A	No	No
Meinero	Video-assisted anal fistula treatment: a novel sphincter-saving procedure for treating complex anal fistulas	2011	Retrospective review	VAAFT	136	3	No	N/A	No	N/A	No	No
Meinero	Video-assisted anal fistula treatment: a new concept of treating anal fistulas	2014	Retrospective review	VAAFT	203	5	No	N/A	No	N/A	No	No
Mennigen	The OTSC proctology clip system for the closure of refractory anal fistulas	2015	Retrospective review	Clip	10	4	Yes	Yes	No	N/A	No	No
Mishra	The role of fibrin glue in the treatment of high and low fistulas in ano	2013	Prospective study	Fibrin glue	30	4	No	N/A	No	N/A	No	No
Mitalas	Does rectal mucosal blood flow affect the outcome of transanal advancement flap repair?	2009	Prospective study	Advancement flap	54	3	No	N/A	No	N/A	Yes	No
Mushaya	Ligation of intersphincteric fistula tract compared with advancement flap for complex anorectal fistulas requiring initial seton drainage	2012	RCT	LIFT procedure Advancement flap	39	6	No	N/A	No	N/A	No	Yes
Nazeer	Better option for the patients of low fistula in ano: fistulectomy or fistulotomy	2012	RCT	Fistulotomy Fistulectomy	150	5	No	N/A	No	N/A	No	No
Nordholm-Carstensen	Treatment of complex fistula-in-ano with nitinol proctology clip	2017	Retrospective review	Clip	35	2	Yes	Yes	Yes	Yes	Yes	No
Omar	Drainage seton versus external anal sphincter-sparing seton after rerouting of the fistula tract in the treatment of complex anal fistula: a randomized controlled trial	2019	RCT	Seton Rerouting of fistula tract and seton around internal anal sphincter	60	6	Yes	Yes	Yes	Yes	No	No
Ommer	Gore BioA fistula plug in the treatment of high anal fistulas - initial results from a German multicenter-study	2012	Retrospective review	AFP	40	1	No	N/A	N/A	N/A	Yes	No
Ortiz	Randomized clinical trial of anal fistula plug versus endorectal advancement flap for the treatment of high cryptoglandular fistula in ano	2009	RCT	AFP ERAF	32	2	Yes	No	Yes	Yes	Yes	No
Ortiz	Length of follow‐up after fistulotomy and fistulectomy associated with endorectal advancement flap repair for fistula in ano	2008	Prospective study	Fistulotomy Fistulectomy and ERAF	206	2	No	N/A	No	N/A	No	No
Owen	Plugs unplugged. Anal fistula plug: the Concord experience	2010	Retrospective review	AFP	32	1	Yes	Yes	N/A	N/A	No	No
Ooi	Managing fistula‐in‐ano with ligation of the intersphincteric fistula tract procedure: the Western Hospital experience	2012	Prospective study	LIFT procedure	25	2	Yes	Yes	Yes	Yes	Yes	No
Ozturk	Treatment of recurrent anal fistula using an autologous cartilage plug: a pilot study	2015	Prospective study	AFP	10	5	No	N/A	No	N/A	Yes	No
Ozturk	Laser ablation of fistula tract: a sphincter-preserving method for treating fistula-in-ano	2014	Retrospective review	Laser ablation	37	1	Yes	Yes	N/A	N/A	Yes	No
Prosst	Short-term outcomes of a novel endoscopic clipping device for closure of the internal opening in 100 anorectal fistulas	2016	Retrospective review	Clip	96	2	No	N/A	No	N/A	No	No
Ratto	Fistulotomy with end-to-end primary sphincteroplasty for anal fistula: results from a prospective study	2013	Prospective study	FISR	72	2	No	N/A	No	N/A	No	No
Roig	Changes in anorectal morphologic and functional parameters after fistula-in-ano surgery	2009	Prospective study	Fistulotomy FISR Seton Fistulectomy and advancement flap	120	4	No	N/A	No	N/A	Yes	No
Roig	Fistulectomy and sphincteric reconstruction for complex cryptoglandular fistulas	2010	Retrospective review	ERAF FISR	146	7	No	N/A	No	N/A	No	No
Safar	Anal fistula plug: initial experience and outcomes	2009	Retrospective review	AFP	35	1	No	N/A	No	N/A	Yes	No
Sanad	A randomized controlled trial on the effect of topical phenytoin 2% on wound healing after anal fistulotomy	2019	RCT	Fistulotomy and phenytoin Fistulotomy	60	6	Yes	Yes	Yes	Yes	No	No
Schulze	Management of complex anorectal fistulas with seton drainage plus partial fistulotomy and subsequent ligation of intersphincteric fistula tract (LIFT)	2015	Prospective study	Seton Fistulotomy and LIFT procedure	75	5	Yes	Yes	N/A	N/A	No	No
Schwandner	Surgical treatment of complex anal fistulas with the anal fistula plug: a prospective, multicenter study	2009	Prospective study	AFP	60	4	No	N/A	No	N/A	No	No
Schwandner	Randomized clinical trial comparing a small intestinal submucosa anal fistula plug to advancement flap for the repair of complex anal fistulas	2018	RCT	AFP Advancement flap	82	5	Yes	Yes	Yes	Yes	No	No
Seneviratne	Quality of life following surgery for recurrent fistula-in-ano	2009	Prospective study	Fistulotomy Fistulectomy Seton	21	1	No	N/A	No	N/A	No	No
Seow-En	An experience with video-assisted anal fistula treatment (VAAFT) with new insights into the treatment of anal fistulae	2016	Retrospective review	VAAFT	41	3	Yes	Yes	N/A	N/A	Yes	No
Shafik	Combined partial fistulectomy and electro-cauterization of the intersphincteric tract as a sphincter-sparing treatment of complex anal fistula: clinical and functional outcome	2014	Prospective study	Fistulectomy and electro-cauterization	53	4	No	N/A	No	N/A	No	No
Shanwani	Ligation of the intersphincteric fistula tract (LIFT): a sphincter-saving technique for fistula-in-ano	2010	Prospective study	LIFT procedure	45	4	No	N/A	No	N/A	No	No
Sileri	Surgery of fistula-in-ano in a specialist colorectal unit: a critical appraisal	2011	Prospective study	Seton Fistulotomy LIFT procedure Advancement flap	247	6	No	N/A	No	N/A	No	No
Sileri	Ligation of the intersphincteric fistula tract (LIFT) to treat anal fistula: early results from a prospective observational study	2011	Prospective study	LIFT procedure	18	4	No	N/A	No	N/A	No	No
Stamos	Prospective multicenter study of a synthetic bioabsorbable anal fistula plug to treat cryptoglandular transsphincteric anal fistulas	2015	Prospective study	AFP	93	5	No	N/A	No	N/A	No	No
Stroumza	Surgical treatment of transsphincteric anal fistulas with the Fat GRAFT technique: a minimally invasive procedure	2017	Prospective study	Fat grafting	11	3	Yes	Yes	Yes	No	Yes	No
Sugrue	Sphincter-sparing anal fistula repair: are we getting better?	2017	Retrospective review	LIFT procedure Fibrin glue Advancement flap AFP Advancement flap and AFP Advancement flap and advancement flap	462	1	Yes	No	N/A	N/A	Yes	No
Shanwari	Ligation of the intersphincteric fistula tract (LIFT): a sphincter-saving technique for fistula-in-ano	2010	Prospective study	LIFT procedure	45	4	Yes	Yes	No	N/A	No	Yes
Schwandner	Initial experience on efficacy in closure of cryptoglandular and Crohn’s transsphincteric fistula by the use of the anal fistula plug	2008	Prospective study	AFP	19	3	Yes	Yes	Yes	Yes	No	No
Sirikurnpiboon	Ligation of intersphincteric fistula tract and its modification: results from treatment of complex fistula	2013	Prospective study	LIFT procedure LIFT procedure and fistulectomy	41	6	No	N/A	No	N/A	No	No
Sungurtekin	Loose seton: a misnomer of cutting seton	2016	Prospective study	Seton	50	3	Yes	Yes	Yes	Yes	No	No
Tan	To LIFT or to flap? Which surgery to perform following seton insertion for high anal fistula?	2012	Retrospective review	ERAF LIFT procedure	31	2	No	N/A	No	N/A	Yes	No
Tan	The anatomy of failures following the ligation of intersphincteric tract technique for anal fistula: a review of 93 patients over 4 years	2011	Retrospective review	LIFT procedure	93	3	No	N/A	No	N/A	Yes	No
Terzi	Closing perianal fistulas using a laser: long-term results in 103 patients	2018	Retrospective review	FiLaC	103	2	No	N/A	No	N/A	No	No
Tobisch	Total fistulectomy with simple closure of the internal opening in the management of complex cryptoglandular fistulas: long-term results and functional outcome	2012	Retrospective review	Fistulectomy and closure of internal opening	252	2	No	N/A	No	N/A	Yes	No
Tokunaga	Clinical role of a modified seton technique for the treatment of trans-sphincteric and supra-sphincteric anal fistulas	2013	Retrospective review	Seton	239	2	No	N/A	No	N/A	Yes	Yes
Tozer	Fistulotomy in the tertiary setting can achieve high rates of fistula cure with an acceptable risk of deterioration in continence	2013	Retrospective review	Fistulotomy	50	3	No	N/A	No	N/A	No	No
Tan	Early experience of reinforcing the ligation of the intersphincteric fistula tract procedure with a bioprosthetic graft (BioLIFT) for anal fistula	2014	Retrospective study	BioLIFT procedure	13	4	No	N/A	No	N/A	No	No
Thekkinkattil	Efficacy of the anal fistula plug in complex anorectal fistulae	2009	Prospective study	AFP	43	2	Yes	Yes	No	N/A	No	No
Tsunoda	Anal function after ligation of the intersphincteric fistula tract	2013	Prospective study	LIFT procedure and seton	20	3	No	N/A	No	N/A	Yes	No
Van Koperen	The anal fistula plug versus the mucosal advancement flap for the treatment of anorectal fistula (PLUG trial)	2008	RCT	AFP Advancement flap	60	4	No	N/A	No	N/A	Yes	No
Van Koperen	Fibrin glue and transanal rectal advancement flap for high transsphincteric perianal fistulas; is there any advantage?	2008	Retrospective study	Fibrin glue and advancement flap	80	2	Yes	Yes	Yes	No	Yes	No
Van Koperen	The anal fistula plug treatment compared with the mucosal advancement flap for cryptoglandular high transsphincteric perianal fistula: a double-blinded multicenter randomized trial	2011	RCT	AFP Advancement flap	60	5	No	N/A	No	N/A	No	No
Van Koperen	Long-term functional outcome and risk factors for recurrence after surgical treatment for low and high perianal fistulas of cryptoglandular origin	2008	Retrospective review	Fistulotomy Advancement flap	179	3	No	N/A	No	N/A	No	No
Van Onkelen	Treatment of anal fistulas with high intersphincteric extension	2013	Retrospective review	Advancement flap and drainage of abscess	14	3	No	N/A	No	N/A	Yes	No
Van Onkelen	Is it possible to improve the outcome of transanal advancement flap repair for high transsphincteric fistulas by additional ligation of the intersphincteric fistula tract?	2012	Prospective study	LIFT procedure and advancement flap	41	2	No	N/A	No	N/A	Yes	No
Van Onkelen	Ligation of the intersphincteric fistula tract in low transsphincteric fistula: a new technique to avoid fistulotomy	2013	Retrospective study	LIFT procedure	22	2	Yes	Yes	No	N/A	No	No
Van Onkelen	Predictors of outcome after transanal advancement flap repair for high transsphincteric fistulas	2014	Retrospective review	Advancement flap	252	2	No	N/A	No	N/A	Yes	No
Visscher	Long-term follow-up after surgery for simple and complex cryptoglandular fistulas: fecal incontinence and impact on quality of life	2015	Retrospective study	Fistulotomy Sphincter-preserving procedures	116	2	No	N/A	No	N/A	Yes	Yes
Walega	VAAFT: a new minimally invasive method in the diagnostics and treatment of anal fistulas-initial results	2014	Prospective study	VAAFT	18	5	No	N/A	No	N/A	No	No
Wallin	Does ligation of the intersphincteric fistula tract raise the bar in fistula surgery?	2012	Retrospective review	LIFT procedure	93	4	No	N/A	No	N/A	No	No
Wang	Traditional Chinese surgical treatment for anal fistulae with secondary tracks and abscess	2012	RCT	Suture dragging and pad compression Fistulotomy	60	6	Yes	No	Yes	No	No	No
Wang	Management of low transsphincteric anal fistula with serial setons and interval muscle-cutting fistulotomy	2016	Retrospective study	Seton Fistulotomy	26	2	Yes	Yes	N/A	N/A	No	No
Wang	Treatment of transsphincteric anal fistulas: are fistula plugs an acceptable alternative?	2009	Retrospective study	AFP Advancement flap	55	1	No	N/A	No	N/A	No	No
Wilhelm	A new technique for sphincter-preserving anal fistula repair using a novel radial emitting laser probe	2011	Retrospective study	FiLaC	11	2	Yes	Yes	Yes	Yes	Yes	No
Wilhelm	Five years of experience with the FiLaC laser for fistula-in-ano management: long-term follow-up from a single institution	2017	Prospective study	FiLaC	117	2	No	N/A	No	N/A	Yes	No
Yan	Clinical effect of tunnel-like fistulectomy plus draining seton combined with incision of internal opening of anal fistula (TFSIA) in the treatment of high-transsphincteric anal fistula	2020	RCT	TFSIA Seton	80	7	No	N/A	No	N/A	No	No
Ye	Early experience with the modificated approach of ligation of the intersphincteric fistula tract for high transsphincteric fistula	2015	Retrospective review	Modified LIFT procedure	43	3	Yes	Yes	Yes	Yes	Yes	No
Yuan	Clinical study on herbal fumigation of detumescence and pain relieving shengji decoction in wound repair after anal fistula surgery	2017	RCT	Shengji decoction Potassium permanganate	90	6	No	N/A	No	N/A	No	No
Zarin	VAAFT: video-assisted anal fistula treatment: bringing revolution in fistula treatment	2015	Prospective study	VAAFT	40	3	Yes	Yes	N/A	N/A	Yes	No
Zubaidi	Anal fistula plug in high fistula-in-ano: an early Saudi experience	2009	Prospective study	AFP	22	1	Yes	No	No	N/A	No	No
Zwiep	Comparison of ligation of the intersphincteric fistula tract and BioLIFT for the treatment of transsphincteric anal fistula: a retrospective analysis	2020	Retrospective review	LIFT BioLIFT	119	4	Yes	Yes	Yes	Yes	No	No
Total					11,819 patients	552 outcomes	65.8%	67.6%	39.7%	64.3%	20.0%	11.0%
Adegbola	Short-term efficacy and safety of three novel sphincter-sparing techniques for anal fistulae: a systematic review	2017	Systematic review									
Alasari	Overview of anal fistula and systematic review of ligation of the intersphincteric fistula tract (LIFT)	2014	Systematic review									
Cirocchi	The treatment of anal fistulas with biologically derived products: is innovation better than conventional surgical treatment? An update	2013	Systematic review									
Cirocchi	Meta-analysis of fibrin glue versus surgery for treatment of fistula-in-ano	2010	Systematic review									
Garg	The efficacy of anal fistula plug in fistula-in-ano: a systematic review	2010	Systematic review									
Hong	Ligation of intersphincteric fistula tract (LIFT) to treat anal fistula: systematic review and meta-analysis	2014	Systematic review									
Jacob	Surgical intervention for anorectal fistula	2010	Systematic review									
Malik	Incision and drainage of perianal abscess with or without treatment of anal fistula	2010	Systematic review									
O’Riordan	A systematic review of the anal fistula plug for patients with Crohn’s and non-Crohn’s related fistula-in-ano	2012	Systematic review									
Pu	Fistula plug versus conventional surgical treatment for anal fistulas: a systematic review and meta-analysis	2012	Systematic review									
Ratto	Fistulotomy or fistulectomy and primary sphincteroplasty for anal fistula (FIPS): a systematic review	2015	Systematic review									
Ritchie	Incontinence rates after cutting seton treatment for anal fistula	2009	Systematic review									
Sirany	The ligation of the intersphincteric fistula tract procedure for anal fistula: a mixed bag of results	2015	Systematic review									
Soltani	Endorectal advancement flap for cryptoglandular or Crohn’s fistula-in-ano	2010	Systematic review									
Vial	Faecal incontinence after seton treatment for anal fistulae with and without surgical division of internal anal sphincter: a systematic review	2010	Systematic review									

*RCT* randomized controlled trial, *ADM* acellular dermal matrix, *ERAF* endorectal advancement flap, *LIFT*  ligation of intersphincteric fistula tract, *EAS* external anal sphincter, *IAS* internal anal sphincter, *AFP* anal fistula plug, *FISR* fistulectomy/fistulotomy and immediate sphincter reconstruction, *EUA* examination under anaesthetic, *MAFT* minimally invasive anal fistula treatment, *ASC* adipose-derived stem cells, *PRP* platelet-rich plasma, *PRGF* plasma-rich growth factor, *PERFACT* proximal superficial cauterization, emptying regularly fistula tracts and curettage of tracts, *FiLaC* fistula laser closure, *VAAFT* video-assisted anal fistula treatment, *PRF* platelet-rich fibrin, *TFSIA* tunnel-like fistulectomy plus draining seton combined with incision of internal opening of anal fistula

### Data synthesis

#### Outcome categorisation

The resulting list of outcomes was reviewed by the study management group, including patient representatives (AM, NI, GK, RW, HG, MK, UG, PT, SB) to enable those with similar wording or meaning to be reduced to a single outcome. These were then mapped according to the COMET taxonomy developed for outcomes in medical research [13]. In this taxonomy, the measurable aspects of health conditions can be structured into five core areas, namely death, physiological or clinical, life impact, resource use, and adverse events, and further subdivided into 38 domains.

#### Data analysis

Primary, secondary, and overall outcome reporting were analysed. Results were summarized using frequencies and percentages. The frequency of outcome domain reporting was calculated. The interventions studied, number of outcome definitions and measurement instruments used were collated and analysed.

## Results

### Search strategy and study selection

**Fig. 1:**
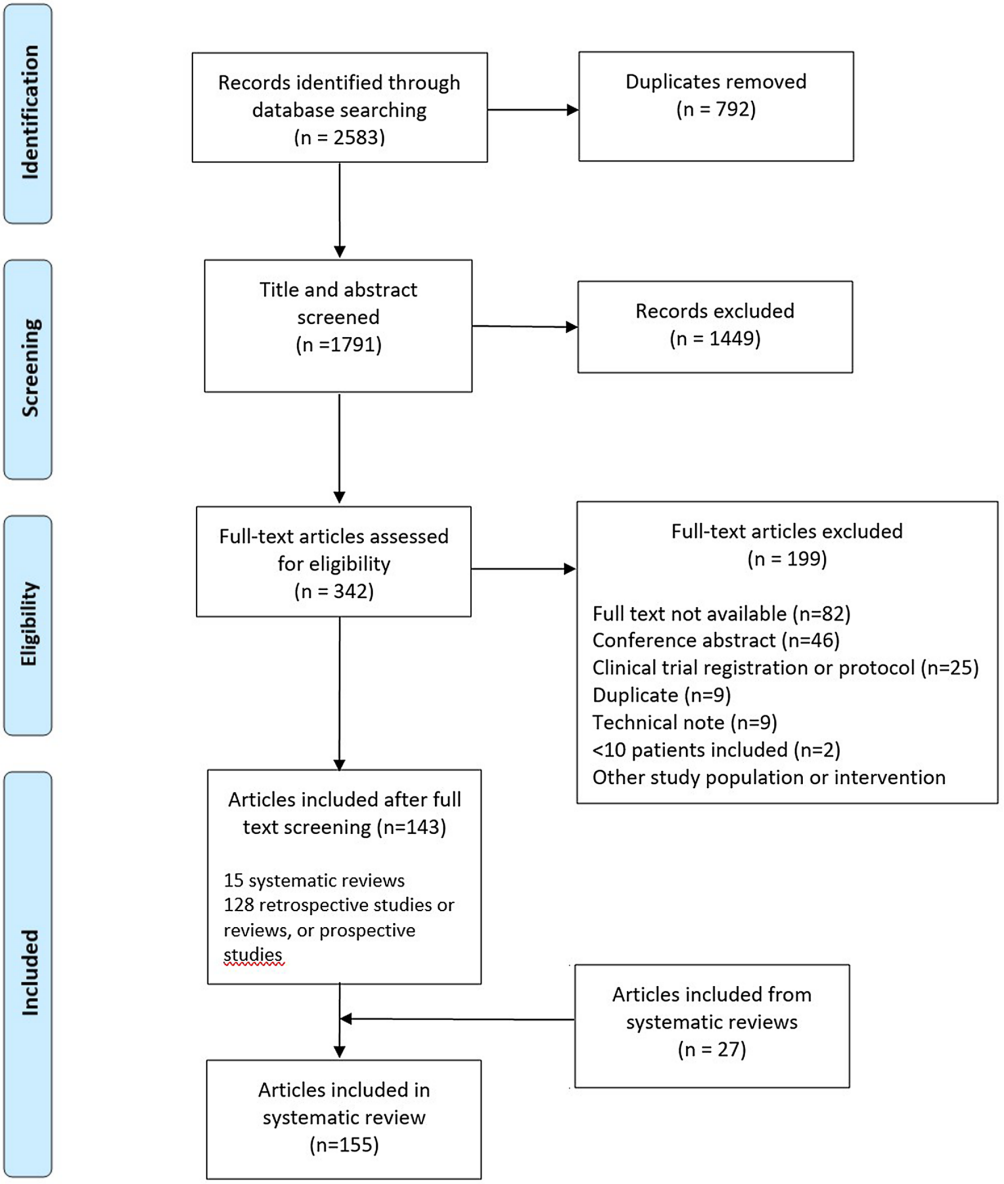
Preferred reporting items for systematic reviews and meta-analyses flow chart of study selection

The electronic databases Medline (Ovid), Embase (Ovid), and The Cochrane Library were searched in May 2018, followed by an updated search in May 2020, identifying a total of 2583 records. A schematic overview of the inclusion and exclusion of articles, including reasons provided for exclusion, is presented in Fig. 1. Full-text screening resulted in the inclusion of 143 articles, including 15 systematic reviews. The systematic reviews were individually screened for any additional studies that were not captured by the initial search and this yielded 27 articles, resulting in a final number of 155 articles from which data were extracted.

### Study characteristics

An overview of the 155 included studies is presented in Table 2. Interventions for cryptoglandular AF were assessed on a total of 11,819 patients (mean 76, range 10–462 participants per study). The majority of studies were prospective studies (52%) and assessed the effectiveness of sphincter-preserving procedures, of which fistula plugs (19%) and ligation of intersphincteric fistula tract (LIFT) procedures (19%) were assessed most frequently. The characteristics of the included studies are presented in Table 3. The quality of outcome reporting for each individual study was assessed using Harman’s criteria [12] and reported in Table 2. The criteria involve assessing whether: (1) The primary outcome for a study is clearly stated, (2) The primary outcome is clearly defined so that other researchers can reproduce its measurement, (3) The secondary outcomes are clearly stated, (4) The secondary outcomes are clearly defined, (5) The authors explain the use of the outcomes they have selected and (6) Any methods were used to enhance the quality of outcome measurement. The average number of criteria met across all studies was two, with only 38 of 155 studies (25%) meeting ≥ four criteria, indicating high-quality outcome reporting in just a quarter of the studies assessed.

**Table 3:** Study characteristics

		*n* (%)
Total included		155 (100)
Study types contributing to data synthesis		
Retrospective studies		66 (43)
Prospective studies *(RCT)*		89 (57) *30 (19)*
Publication year		
2008–2010		40 (25)
2011–2013		50 (32)
2014–2016		35 (23)
2017–2019		27 (17)
2020		3 (2)

Intervention(s)	Control(s)	*n* (%)
AFP	No control Advancement flap ERAF Fistulotomy or cutting seton or advancement flap or LIFT	30 (19)
LIFT LIFT-plug BioLIFT Modified LIFT LIFT and seton LIFT and advancement flap LIFT and fistulectomy	No control LIFT-plug Advancement flap LIFT and fistulectomy BioLIFT	29 (19)
Seton	No control Seton(s) Fistulotomy Fistulotomy and LIFT Rerouting of fistula tract and seton around internal anal sphincter	12 (8)
Fistulotomy Fistulotomy and sucralfate Partial fistulotomy and seton Fistulotomy and phenytoin	No control Advancement flap Fistulectomy Fistulotomy and placebo sucralfate Fistulectomy and ERAF Fistulectomy and seton Fistulotomy and marsupialization Fistulotomy	10 (7)
Advancement flap Advancement flap and platelet-rich plasma Advancement flap and drainage of abscess Advancement flap and drainage seton around external anal sphincter	No control Advancement flap Seton around internal anal sphincter	9 (6)
VAAFT	No control	8 (5)
Fibrin glue Fibrin glue and advancement flap	No control Seton Fibrin glue and ASC	6 (4)
Fistulectomy Fistulectomy and electro-cauterization Fistulectomy and closure of internal fistula opening TFSIA	No control Fistulotomy Fistulotomy and marsupialization Seton	6 (4)
FISR	No control	5 (3)
FiLaC or laser ablation	No control	5 (3)
Clip	No control Fistulectomy	5 (3)
ERAF	AFP LIFT FISR	3 (2)
ASC ASC and fibrin glue	No control ASC, fibrin glue, and fibrin glue Fibrin glue	3 (2)
ADM	No control ERAF	2 (1)
Modified Hanley Modified Hanley, drainage, and setons	No control	2 (1)
Collagen paste injection	No control Fibrin glue	2 (1)
PRGF	No control	1 (1)
PERFACT procedure	No control	1 (1)
PRF	No control	1 (1)
MAFT	No control	1 (1)
Irrigation and silver nitrate	No control	1 (1)
PRP	Fibrin glue	1 (1)
≥ 4 interventions compared		7 (5)
Other		5 (3)
Total		155 (100)

*AFP* anal fistula plug, *ERAF* endorectal advancement flap, *LIFT* ligation of intersphincteric fistula tract, *VAAFT* video-assisted anal fistula treatment, *ASC* adipose-derived stem cells, *TFSIA* tunnel-like fistulectomy plus draining seton combined with incision of internal opening of anal fistula, *FISR* fistulectomy/fistulotomy and immediate sphincter reconstruction, *FiLaC* fistula laser closure, *ADM* acellular dermal matrix, *PRGF* plasma-rich growth factor, *PERFACT* proximal superficial cauterization, emptying regularly fistula tracts and curettage of tracts, *PRF* platelet-rich fibrin, *MAFT* minimally invasive anal fistula treatment, *PRP* platelet-rich plasma

### Study outcomes

In total, 552 patient- and clinician-reported outcomes were extracted from 155 studies, with studies reporting a median of three outcomes (interquartile range 2–5) per study. Duplicate and analogous terms were merged to form 52 outcomes, of which healing (77%), incontinence (63%), recurrence (40%), and pain (26%) were reported most frequently (Table 4). Outcomes such as healing and recurrence were sometimes measured at different time points within the same study but referred to as primary or secondary outcomes. This resulted in some studies reporting outcomes of healing and recurrence more than once.

**Table 4:** Frequency of outcome reporting

Outcome	Reported as primary outcome (*n*)	Reported as secondary outcome (*n*)	Unstated (*n*)	Number of studies reporting outcome (*n*) (%)
Healing	80	12	38	120 (77)
Incontinence	24	35	39	98 (63)
Recurrence	16	21	27	62 (40)
Pain	12	16	12	40 (26)
Complications	15	10	11	36 (23)
Closure time	13	3	9	25 (16)
Treatment failure	4	4	14	22 (14)
Quality of life	6	12	4	22 (14)
Duration of treatment	4	7	1	12 (8)
Morbidity	1	3	7	11 (7)
Return to work	2	2	3	7 (5)
Patient satisfaction	2	2	3	7 (5)
Anorectal manometry	0	0	6	6 (4)
Pus discharge	2	3	0	5 (3)
Hospital stay	1	2	2	5 (3)
Adverse effects	2	3	0	5 (3)
Fistula persistence	3	1	0	4 (3)
Reinterventions	0	1	3	4 (3)
Safety	1	3	0	4 (3)
Plug dislodgement rates	1	2	0	3 (2)
Symptoms	3	0	0	3 (2)
Unit cutting time	2	0	0	2 (1)
Size of operative wound	0	2	0	2 (1)
Postoperative perineal sepsis	1	0	1	2 (1)
Postoperative bleeding	0	0	2	2 (1)
Cost-effectiveness	1	1	0	2 (1)
Difficulty of technique	2	0	0	2 (1)
Impact on daily life	0	2	0	2 (1)
Endoanal ultrasound	0	0	2	2 (1)
Radiological healing	0	1	0	1 (1)
Anorectal deformity rate	0	1	0	1 (1)
Burning sensation	0	1	0	1 (1)
Itching	0	1	0	1 (1)
Length of time until seton removal	0	1	0	1 (1)
Fraction of patients showing ≥ 50% decrease in fistula size	0	1	0	1 (1)
Investigator’s satisfaction score	0	1	0	1 (1)
Amount of mucosal covering	0	1	0	1 (1)
Asymptomatic	1	0	0	1 (1)
Subjective parameters	0	1	0	1 (1)
Glue reaction	0	0	1	1 (1)
Median mucosal blood flow	0	0	1	1 (1)
Problems related to sexual function	0	0	1	1 (1)
Pudendal nerve terminal motor latency	0	0	1	1 (1)
Duration for return to normal activity	1	0	0	1 (1)
Duration of immobilisation	1	0	0	1 (1)
Emerging of a secondary abscess	1	0	0	1 (1)
Need for a new wave of drainage	1	0	0	1 (1)
Removal or migration of the clip	1	0	0	1 (1)
Perianal incision wound healing	1	0	0	1 (1)
Analgesic requirement	0	0	1	1 (1)
Keyhole like anomaly	0	0	1	1 (1)
Conversion into intersphincteric perianal fistula	0	1	0	1 (1)

### Outcome categorisation

The outcomes were categorized into core areas and domains according to the COMET taxonomy, with guidance from a member of COMET. The frequency of these outcomes and their categorisation is shown in Table 5. Adverse event outcomes are categorised under their appropriate taxonomy and identified as a harm outcome [13]. Cryptoglandular AF treatment rarely impacts lifespan, therefore the core area death was excluded from categorisation. Some outcomes were categorised in multiple domains, as the study management group considered their impact to be broad. For instance, ‘problems related to sexual function’ was included in the domains physical, social and emotional functioning and well-being. Outcomes belonging to the core area of ‘physiological or clinical’ were placed in domains according to their underlying cause or affected body system [13]. Whilst categorisation highlighted the spread of outcomes across all relevant domains, the majority focused on the physiological or clinical impact, particularly in the domain of gastrointestinal outcomes (99%), whereas only 12% of outcomes were related to the impact on physical, role and social functioning and emotional functioning and wellbeing (Table 5).

**Table 5:** Outcome categorisation and frequency of outcome reporting according to the COMET taxonomy

Core area	Domain	Outcomes	No. studies reporting outcomes (%)
Physiological or clinical	Gastrointestinal outcomes	Healing	154 (99)
		Incontinence (harm)	
		Recurrence (harm)	
		Pain	
		Treatment failure (harm)	
		Closure time	
		Pus discharge	
		Anorectal manometry	
		Fistula persistence (harm)	
		Plug dislodgement rates (harm)	
		Unit cutting time	
		Symptoms	
		Radiological healing	
		Anorectal deformity rate (harm)	
		Burning sensation	
		Itching	
		Fraction of patients showing ≥ 50% decrease in fistula size	
		Amount of mucosal covering	
		Asymptomatic	
		Subjective parameters	
		Glue reaction (harm)	
		Endoanal ultrasound	
		Pudendal nerve terminal motor latency	
		Removal or migration of the clip (harm)	
		Perianal incision wound healing	
		Conversion into intersphincteric fistula (harm)	
		Keyhole like anomaly (harm)	
	General outcomes	Morbidity (harm)	11 (7)
	Infection and infestation outcomes	Postoperative perineal sepsis (harm)	3 (2)
		Emerging of a secondary abscess (harm)	
	Vascular outcomes	Median mucosal blood flow	2 (1)
		Postoperative bleeding (harm)	
Life impact	Physical functioning	Problems related to sexual functioning	5 (3)
		Duration for return to normal activity	
		Duration of immobilisation	
		Impact daily life	
	Social functioning	Problems related to sexual functioning	3 (2)
		Impact daily life	
	Role functioning	Return to work	8 (5)
		Impact daily life	
	Emotional functioning or well-being	Problems related to sexual functioning	3 (2)
		Impact daily life	
	Global quality of life	Quality of life	22 (14)
	Delivery of care	Treatment failure	32 (22)
		Duration of treatment	
		Patient satisfaction	
		Size of operative wound	
		Length of time until seton removal	
		Investigator’s satisfaction score	
		Difficulty of technique	
Resource use	Economic	Cost-effectiveness	2 (1)
	Hospital	Hospital stay	5 (3)
	Need for further intervention	Reinterventions	6 (4)
		Need for a new wave of drainage	
		Analgesic requirement	
Adverse events	Adverse events and/or effects	Complications	44 (28)
		Adverse effects	
		Safety	

### Outcome definitions

Significant heterogeneity in outcome definition and overlap between definitions was noted in the outcomes of ‘healing’, ‘recurrence’, and ‘treatment failure’.

#### Healing

Healing was reported in 120 studies (77%) and was synonymous with terms such as ‘healing rate’, ‘fistula closure’, ‘success’, ‘cure’, ‘effectiveness’, and ‘complete clinical response’. There was considerable heterogeneity in the definitions of healing, however, overlap between the components of each definition meant that all could be defined by using one or more of the components presented in Table 6. Considering the ways in which components could be combined, 34 different definitions were found. Healing was most frequently defined as ‘healing of the external fistula opening and absence of symptoms’ (*n* = 16). In nine studies, a radiological assessment was needed to confirm or refute healing [14–22], whereas another study identified ‘radiological healing’ as a separate outcome [23]. Five of these 10 studies included the radiological description required to demonstrate healing [14, 15, 18, 21, 22]. In 21 studies, the definition of healing was dependent upon a time period after which the fistula should be assessed, or for the duration of which the components of healing should be present, which in themselves demonstrated significant variation, ranging from 2 weeks [24] to 12 months [16, 25] after the procedure.

**Table 6:** Components used, in varying combinations, to define the outcome ‘healing’

Component	Times used
Absence of symptoms	70
Closure of the external fistula opening	61
Absence of abscess or infection or inflammation or sepsis	27
Closure of the (surgical) wound	24
Closure of the internal fistula opening	15
Closure of the fistula tract	14
No additional intervention required	8
Absence of recurrence or persistence or treatment failure	8
Absence of anal sphincter injury	1

#### Recurrence, treatment failure and persistence

The terms recurrence, treatment failure, and persistence were used interchangeably to describe a spectrum of clinical manifestations, ranging from no evidence of closure or persistence of fistula and symptoms [26–29], to temporary closure followed by re-appearance of the original fistula [26], to the development of additional fistulas [20, 30–32]. Similar to healing, the definitions were broken down into components which are presented in Table 7. The most frequently used definitions were ‘persistence or recurrence of symptoms’ (*n* = 21), followed by ‘persistence or reappearance of the external fistula opening’ (*n* = 13). There were 19 different definitions of recurrence and treatment failure. In 10 studies, the definition was qualified by a time period at or after which the fistula had to be assessed**,** ranging from within the first month [20] to 12 months after treatment [33].

**Table 7:** Components used, in varying combinations, to define the outcomes ‘recurrence’ and ‘treatment failure’

Component	Times used
Persistence or recurrence of symptoms	21
Reappearance of the fistula after healing	16
Persistence or reappearance of the external fistula opening	13
Absence of wound healing	8
Abscess or infection	6
Absence of fistula closure or persistence	6
Non-healing fistula	3
Additional intervention required	3
Additional fistula	2

### Outcome measurement instruments

Heterogeneity was noted amongst the measurement instruments used for the most frequently reported outcomes (Table 8). Combinations of measurement instruments were frequently used. Furthermore, the instruments for each outcome were not always clearly stated and many studies used unspecified questionnaires.

**Table 8:** Measurement instruments used, in varying combinations, to assess the most frequently reported outcomes

Outcome	Instruments (used in various combinations)	Times used
Healing	Clinical examination, including digital rectal examination	88
	(Telephone) interview	16
	MRI	9
	(3D) endoanal ultrasound	7
	Medical record review	7
	Anoscopy or proctoscopy or rectoscopy	7
	(Un)specified questionnaire	6
	Digital photograph of the external fistula opening	2
	Transanal ultrasound	1
	Examination under anaesthetic	1
	Anal endosonography	1
Incontinence/sphincter function	Wexner Cleveland Clinic Florida incontinence score	48
	Patient-reported	9
	Vaizey incontinence score	9
	Fecal Incontinence Quality of Life Scale	6
	(Un)specified questionnaire	5
	Anorectal manometry	5
	Endoanal ultrasound	4
	Specified grading system	3
	Clinical examination, including digital rectal examination	3
	Colorectal functional outcome questionnaire	3
	(Telephone) interview	3
	Medical record review	2
	German Society of Coloproctology score	1
	Williams grade	1
	Fecal Incontinence Severity Index	1
Recurrence Treatment failure	Clinical examination	43
	(Telephone) interview	7
	MRI	6
	Medical record review	6
	(Un)specified questionnaire	3
	Endorectal ultrasound	2
	Anoscopy or proctoscopy	2
	3D endoanal ultrasound	1
	Anal endosonography	1
	Patient-reported	1
Quality of life	Fecal Incontinence Quality of Life Scale	6
	Short Form-36 health survey (SF-36)	6
	EQ-5D	4
	Short Form-12 health survey (SF-12)	2
	Cleveland global quality of life	2
	Gastrointestinal Quality of Life Index	2
	(Un)specified questionnaire	2
	Quality of Life Scale	1
	Visual Analogue Scale (VAS)	1
	Fecal Incontinence Severity Index	1
Pain	VAS	31
	Patient-reported	2
	Specified grading system	1
	Medical record review	1
	Number of analgesics used	1

*MRI* magnetic resinance imaging, *VAS* Visual Analogue Scale, *EQ-5D* EuroQol five-dimensions questionnaire

## Discussion

This systematic review is the first study to provide an overview of the outcomes reported in interventional studies for AF. We identified 552 outcomes from 155 studies published in the last 12 years, which were merged into 52 unique outcomes, of which healing was reported most frequently (77%). Our results demonstrate heterogeneity in outcome definition and measurement, making the use of such studies to supplement current understanding of fistula management and guide treatment pathways much more challenging.

The lack of consistency and clarity in definitions of success, treatment failure, and recurrence after fistula treatment has been previously noted [34]. Despite being one of the most frequently reported outcomes, healing was variably defined in terms of anatomical features, absence of a specific set of symptoms or healing of the (surgical) wound. This highlights the difficulty of data synthesis across different studies, particularly when a fistula has healed in one study simply by closure of the external fistula opening [35], but would be considered persistent in another, where both the external and internal fistula openings, and an absence of symptoms are required [36]. The addition of radiological healing provides additional complexity, as it is well documented that deep tissue healing of perianal fistula as assessed on magnetic resonance imaging lags behind clinical healing by a period of months [37–39]. Nevertheless, radiological outcomes and objective measures of the disease have been frequently used in studies of AF, and their potential inclusion in a COS warrants further discussion and involvement of radiological expertise.

The various definitions of recurrence, persistence, and treatment failure demonstrated overlap, however, in line with previous suggestions [34], we determined that treatment failure and persistence of the fistula, i.e. no change in the morphology and symptomatology of the original fistula, should be differentiated from fistula recurrence, which describes reappearance of the fistula after a period of resolution, and that development of new fistulas should be considered separately. However, persistence and recurrence of fistulas could simply be the same problem viewed at different time points, and from a patient’s perspective 1 year after the intervention, the difference is probably minimal. This would be an interesting area to explore during the generation of the COS.

The quality of studies eligible for data extraction was assessed using Harman’s criteria [12], however, only a quarter of the studies demonstrated high-quality outcome reporting using this method. Whilst the majority of studies clearly stated their measured outcomes, few went as far as defining whether the outcomes were primary or secondary. Only 20% of the studies explained their reasoning for selecting their outcomes. This may be due to the fact that healing, incontinence, and recurrence, the most commonly reported outcomes, require little explanation for their selection to fistula surgeons or patients, as the ultimate aim of any fistula treatment is frequently cited as healing with minimal impact on continence, and minimal risk of recurrence.

The outcomes summarised in this systematic review were categorised according to the COMET taxonomy. Although all relevant domains are represented, the vast majority of outcomes are related to the pathophysiology of disease and treatment. Only 10% of the outcomes reported by all studies in the last 12 years were related to the impact of disease in terms of its influence on patients’ physical, social and role functioning, in other words their quality of life. Whilst the inclusion of outcomes such as these is encouraging and should be recognised, their use is infrequent and gives a narrow reflection of the wide-ranging impact that fistula symptoms or treatments have for patients. For example, whilst the impact on sexual functioning has been recognised, the wider effects on personal and social relationships have not been recorded, as well as the influence of symptoms on non-work-related activities. Whilst the pathophysiological aspects of the disease are inevitably interrelated with life impact and use of resources, focusing only on the physical symptoms fails to address adequately the wider impact of living with AF. Earlier studies have identified that patients and surgeons allocate importance to different aspects of quality of life associated with anal fistula and its treatment. Surgeons rated continence, leakage, pain, cure and sepsis, whereas patients identified independent activity, good health, pain, continence, psychological health and leakage as their most important aspects of quality of life [40]. We are currently conducting further qualitative work to explore patients’ experiences of disease further, and patient involvement in deciding the final COS and how these outcomes should be prioritised is crucial to ensure that the COS remains representative of all stakeholders [7] and centred around relevance to patients.

The current study reported the range of outcome measurement instruments used for the most frequently reported outcomes. Validated measures were largely used for outcomes such as incontinence and quality of life, allowing the benefit of comparison across studies, as well as with other chronic health conditions [41]. However, the broad range of validated measures across studies for AF makes it difficult to compare these specific outcomes across interventions. This supports the need for a systematic method of selecting appropriate Outcome Measurement Instruments (OMIs) once the final COS is established [7, 42]. Furthermore, most measurement instruments of quality of life were generic. Disease-specific measures are known to be more sensitive to change and can directly detect the specific concerns of particular clinical groups, which may be underrepresented in generic measurement instruments [43]. Planned qualitative work will help to determine whether the concerns of patients with AF are adequately addressed by these instruments, or whether the development of a disease-specific Patient-Reported Outcome Measure (PROM) is needed.

The strength of this systematic review is that with the range of studies reviewed, it is well placed to inform a long list of items for the development of a COS. However, it is limited by the lack of outcomes related to the quality of life, suggesting that the additional qualitative feedback from patients required by COMET to supplement this longlist is crucial. Although it is possible that not all relevant studies have been captured due to the eligibility criteria used, the sheer number of outcomes extracted from the included studies make it likely that saturation has been reached and that any additional outcomes would be procedure specific, and, therefore, not eligible for a generic COS representing a minimum set of outcomes to be adopted by all studies, regardless of intervention used. A further limitation is the English language inclusion criterion, although no abstracts or full texts were excluded based on the language criterion alone, rather they studied the wrong population or were review articles or commentaries. The lack of non-English papers may limit the generalisability of these findings across cultural and ethnic groups. This may be effectively countered through the subsequent longlisting and consensus processes, which will include a broad ethnic and cultural diversity.

## Conclusions

This systematic review highlights the need for consensus amongst researchers and clinicians regarding the outcomes that are essential in determining successful fistula treatment, and how they should be defined and measured. The underrepresentation of outcomes relating to the quality of life needs to be challenged, and qualitative exploration of the patient experience, as well as active engagement of patients in determining a COS are crucial.

## Data Availability

Registered protocol is available on Prospero (CRD42018102778).
